# Comparison of pleural drain amylase and serum C-reactive protein for early detection of intrathoracic esophago-gastric anastomotic leaks

**DOI:** 10.1007/s00423-022-02550-4

**Published:** 2022-05-17

**Authors:** Erika Andreatta, Alberto Buogo, Emanuele Asti, Sara Boveri, Luigi Bonavina

**Affiliations:** Department of Biomedical Sciences for Health, Division of General and Foregut Surgery, IRCCS Policlinico San Donato, University of Milan, Via Morandi 30, San Donato Milanese, 20097 Milan, Italy

**Keywords:** Esophagectomy, Anastomotic leak, Serum C-reactive protein, Pleural drain amylase

## Abstract

**Introduction:**

Early detection of anastomotic leaks following esophagectomy has the potential to reduce hospital length of stay and mortality. The aim of this study was to compare the predictive value of pleural drain amylase and serum C-reactive protein for the early diagnosis of leak.

**Methods:**

A retrospective observational cohort study was conducted on 121 patients who underwent Ivor Lewis esophagectomy and intrathoracic gastric conduit reconstruction. Pleural drain amylase levels were measured daily until postoperative day (POD) 5 and compared with CRP values measured on POD 3, 5, and 7. Specificity and sensitivity for both tests, and the respective ROC curves, were calculated.

**Results:**

Anastomotic leak occurred in 12 patients. There was a significant statistical association between pleural drain amylase and serum CRP levels and the presence of anastomotic leakage. Pleural drain amylase cutoff of 209 IU/L on POD 2 yielded a sensitivity of 75% and a specificity of 94% (AUC = 0.813), whereas CRP cutoff value of 22.5 mg/dL on POD 3 yielded a sensitivity of 56% and a specificity of 92% (AUC = 0.772). The negative likelihood ratio of pleural drain amylase was 0.27 and 0.12 on POD 2 and 5, respectively. There was no statistically significant difference between ROC curves of amylase and CRP on POD 3 and 5 (*p* = 0.79 and *p* = 0.14, respectively).

**Conclusions:**

Pleural drain amylase seems more efficient than serum CRP for early detection of esophago-gastric anastomotic leak. The practice of monitoring drain amylase and CRP may allow safer implementation of enhanced postoperative recovery pathway.

## Introduction

Anastomotic leakage following esophagectomy is a potentially lethal complication which occurs in about 10% of the patients and is often unpredictable. Contrast esophagram is the most commonly used method for identification of anastomotic leaks, but lacks sensitivity (44%), while the specificity is around 95% [1–3]. Endoscopy and CT scan represent second-line investigations in patients with clinically suspected anastomotic leaks and when contrast esophagram is not conclusive. Ideally, less invasive methods, such as serum C-reactive protein (CRP) [4] sampling, may discriminate patients with high suspicion of esophageal leak who need to be excluded from fast-track and enhanced recovery program protocols and require prompt endoscopic or radiological evaluation to confirm the diagnosis. Sampling of pleural fluid drain amylase has been proposed as a screening method to identify patients at high risk of anastomotic leak, but the evidence remains limited [5, 6].

The primary study aim was to assess the predictive value of pleural drain amylase levels for the early diagnosis of anastomotic leakage by establishing the postoperative day (POD) and the cutoffs at which these parameters show the highest diagnostic accuracy.

## Materials and methods

A retrospective, observational single-center study was conducted. Consecutive patients undergoing hybrid, fully minimally invasive, or open Ivor Lewis esophagectomy with intrathoracic esophago-gastric anastomosis were included in the study. The data were prospectively collected, anonymized, and entered into a dedicated database. The study protocol was approved by the internal review board (PSD Protocol 47, 25/11/2019).

Most surgical procedures were hybrid, and included a 2-stage laparoscopic and a serratus-sparing right thoracotomy approach. A standard D2 lymphadenectomy was performed and a 4-cm wide gastric conduit was constructed by sequential firings of EndoGIA stapler. For the thoracic procedure, the right lung was excluded using a double lumen tube or an endobronchial blocker under bronchoscopic guidance. An infracarinal lymphadenectomy was routinely performed. The esophago-gastric anastomosis was performed at or above the level of the arch of the azygos vein using a 25-mm EEA stapler [7]. The pleura was drained using a Blake tube with the tip positioned at the apex of the chest and connected to a J-Vac reservoir exiting the subxiphoid laparoscopic port site [8]. A thorough irrigation of the pleural cavity with 1 liter of saline solution was performed at the end of the procedure. After surgery, patients were kept fasting, and only sips of water were permitted until a Gastrografin swallow study was performed, usually on POD 4. Anastomotic leakage was diagnosed by Gastrografin swallow study, CT scan with oral and intravenous contrast, endoscopy, and/or methylene blue test. The severity of anastomotic leakage was graded according to the Clavien-Dindo classification [9]. Sampling of pleural drain amylase was performed daily on POD 1, 2, 3, 4, and 5. Serum CRP testing was performed on POD 3, 5, and 7. Patients were stratified into two groups according to the presence or absence of anastomotic leaks.

### Statistical analysis

Categorical variables were described as proportions and percentages while continuous variables were presented as means ± standard deviation for normal distributions or median and interquartile range for no normal distributions. Categorical variables in the leak and no leak groups were compared using χ2 tests or Fisher’s exact test as appropriate. Normality of continuous variables was assessed with the Shapiro‐Wilk test and was compared with the *t* test or with the nonparametric Kruskal–Wallis test. Independent comparison of median of serum CRP and pleural amylase between leak and no leak groups, at different time, was analyzed by nonparametric Kruskal–Wallis test.

The performance of serum CRP and pleural drain amylase was evaluated with univariate logistic regression model and the receiver operating characteristic (ROC) curves; comparisons between ROC curves were analyzed by the chi-square test. The accuracy of the model was determined by the area under the ROC curve that it is a measure of the model’s ability to discriminate patients with leak outcome vs no leak. Values from 0.5 (no discrimination beyond chance) to 1.0 (perfect discrimination) were used. The ROC curve, which illustrates sensitivity against false positive rate, was used to obtain optimal cutoff values, sensitivity, specificity, likelihood ratio (LR), and predictive value (PV). Box plots of serum CRP and pleural amylase by time and leak group were also produced. All analyses were performed using SAS 9.4 (SAS Institute, Inc, Cary, NC, USA).

## Results

A total of 121 patients who underwent esophagectomy between November 2016 and March 2020 were included. The incidence of anastomotic leak was 9.9% (12/121). There were no statistically significant differences in the two patients’ groups regarding demographics and clinical/pathological characteristics (Table 1). The duration of the surgical procedure was significantly higher in patients who developed a leak (*p* = 0.03). The length of hospital stay was longer in patients with anastomotic leak (*p* < 0.0001). The median output from the pleural drain was 410 mL (IQR 60) and 390 mL (IQR 60) in patients with and without anastomotic leak, respectively (*p* > 0.73).

**Table 1:** Pre-operative patients’ characteristics, type of surgical procedure, staging, and length of hospital stay in the two groups

Variables	Leak (*n* = 12)		No leak (*n* = 109)		*p*	
Male, *n (%)*	12 (100)		81 (74.3)		0.07	
Age, years, median (IQR)	63.5 (19.0)		65.0 (15.0)		0.51	
BMI, kg/m^2^, median (IQR)	24.8 (5.1)		24.9 (5.3)		0.77	
Smokers, *n (%)*	7 (58.3)		52 (48.2)		0.56	
Comorbidities, *n (%)*						
Arterial hypertension	5 (45.5)		47 (43.5)		1.00	
Coronary artery disease	1 (9.1)		9 (8.3)		1.00	
Diabetes	1 (9.1)		15 (13.9)		1.00	
Dyslipidemia	-		21 (19.4)		0.21	
Arrhythmia	-		10 (9.2)		0.60	
Liver steatosis	2 (18.2)		1 (0.9)		0.02	
COPD	1 (9.1)		9 (8.3)		1.00	
Histology, *n (%)*					0.85	
Adenocarcinoma	7 (63.6)		79 (76.7)			
Squamous cell carcinoma	1 (9.0)		13 (12.6)			
Other	3 (27.3)		11 (10.6)			
Neoadjuvant therapy, *n (%)*	4 (33.3)		52 (48.2)		0.33	
Ivor Lewis esophagectomy					0.38	
Open	-		1 (0.9)			
Hybrid	7 (58.3)		90 (82.6)			
Totally mini-invasive	5 (41.7)		18 (16.5)			
Operative time, min., median (IQR)	422.5 (172.5)		360.0 (90.0)		0.03	
*p* stage, *n (%)*					0.12	
0	1 (0.1)		7 (6.8)			
I	-		21 (20.4)			
II		4 (0.4)		19 (18.5)		
III		4 (0.4)		37 (35.6)		
IV		1 (0.1)		19 (18.5)		
Length of stay, days, median (IQR)	33.5 (38.5)		11.0 (2.5)		< 0.0001	

**Fig. 1:**
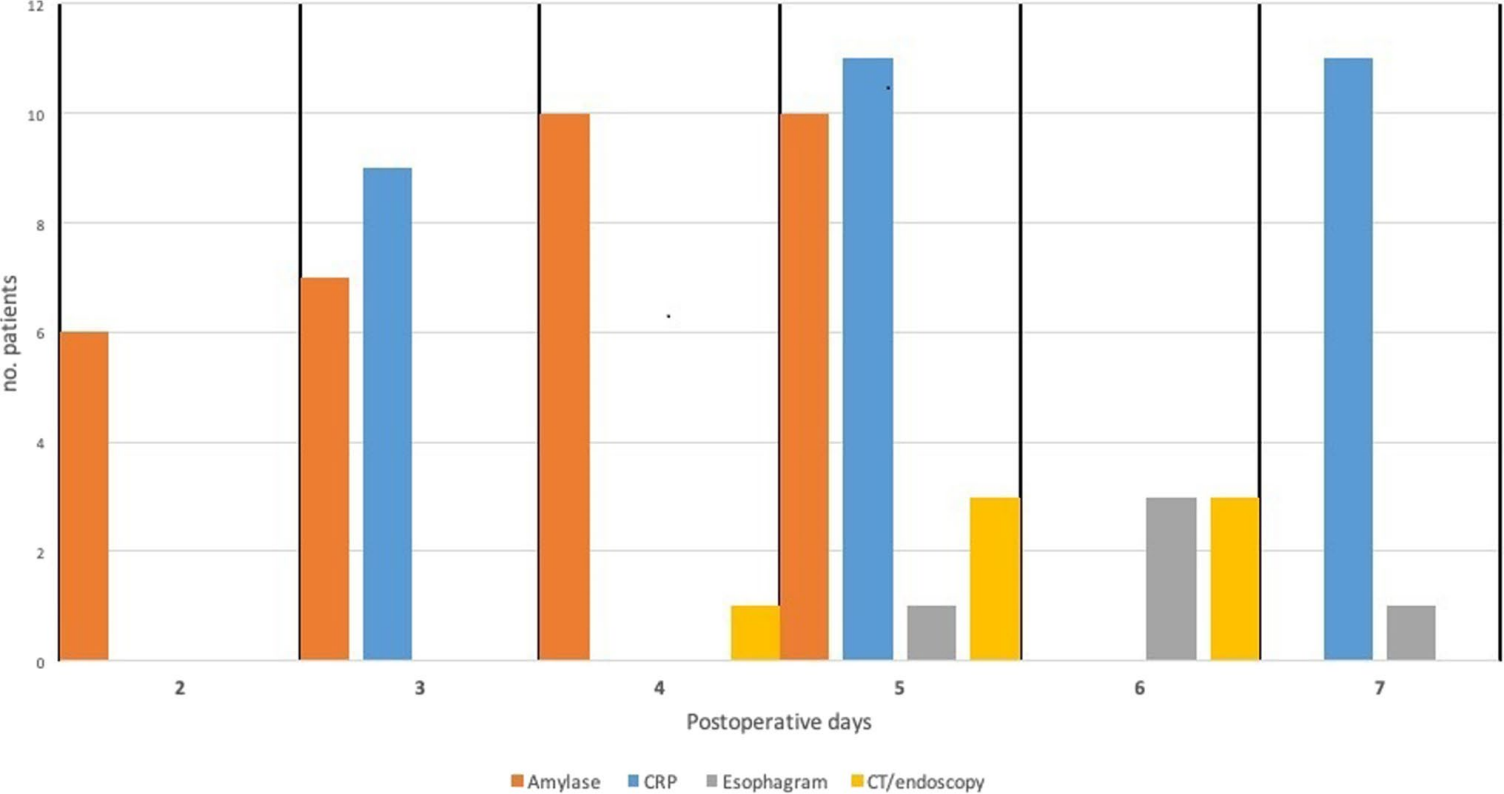
Timeline of positive diagnostic tests in 12 patients with documented anastomotic leak

Six of the 12 patients with anastomotic leak were asymptomatic, and one of them had a positive methylene blue test on POD 5. The remaining patients presented with low-grade fever (*n* = 3) and/or dyspnea (*n* = 3). According to the Clavien-Dindo classification, 3 patients had grade 3a leak, 8 grade 3b, and 1 grade 4a. Figure 1 shows the timeline of all positive diagnostic tests in patients with documented leak. The median time to diagnosis of leak was 5 days (POD range 4–7). Treatment consisted of endoVAC (*n* = 4), thoracoscopic drainage + stent (*n* = 4), stent (*n* = 3), fasting, antibiotics, and naso-jejunal feeding (*n* = 3). There was one hospital death (1/12, 8.3%). The median length of hospital stay was 35.5 days (IQR 34.75).

**Fig. 2:**
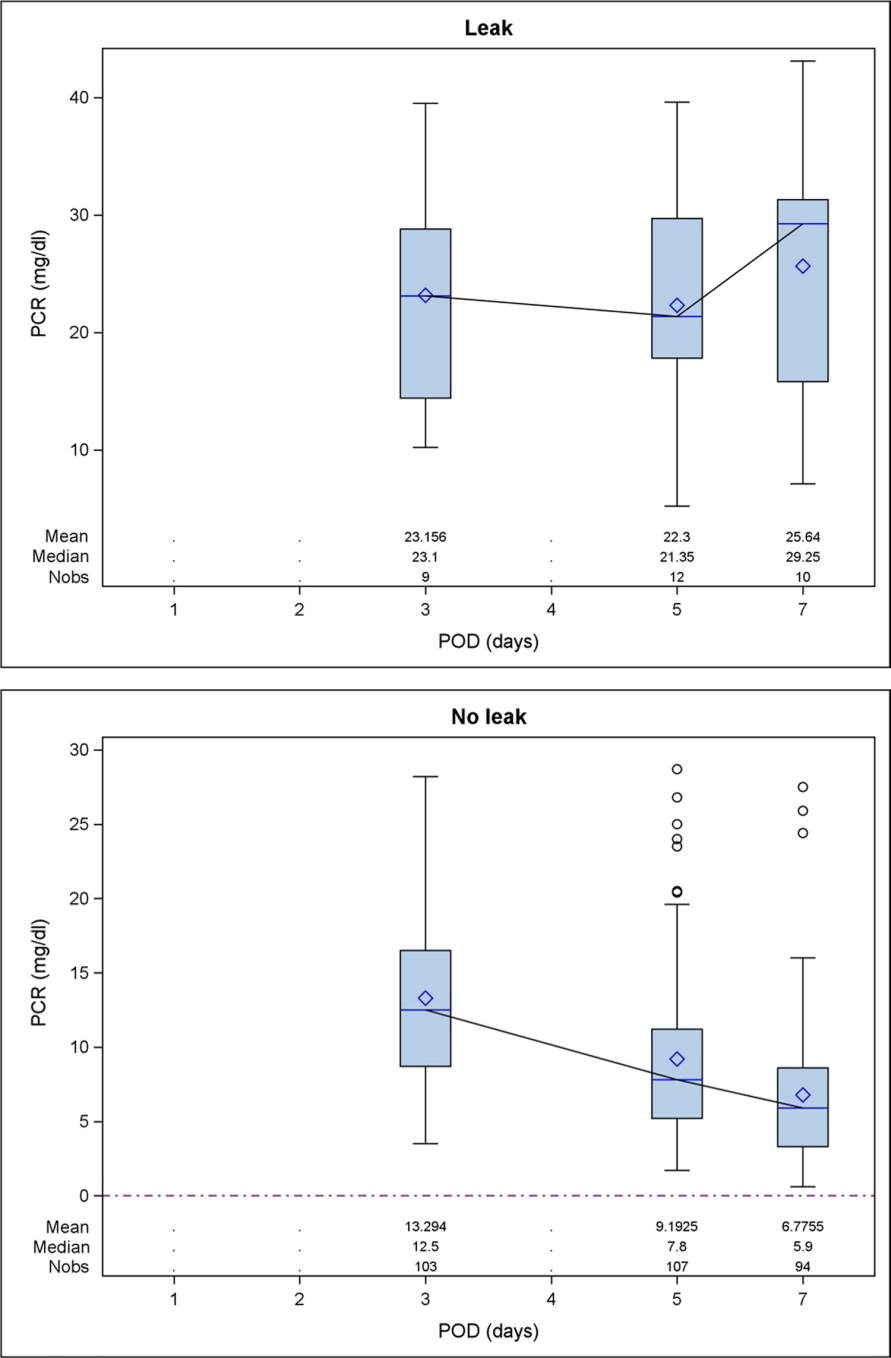
Postoperative variability of C-reactive protein (CRP) serum levels in the patient groups (no leak vs leak). POD, postoperative day

**Fig. 3:**
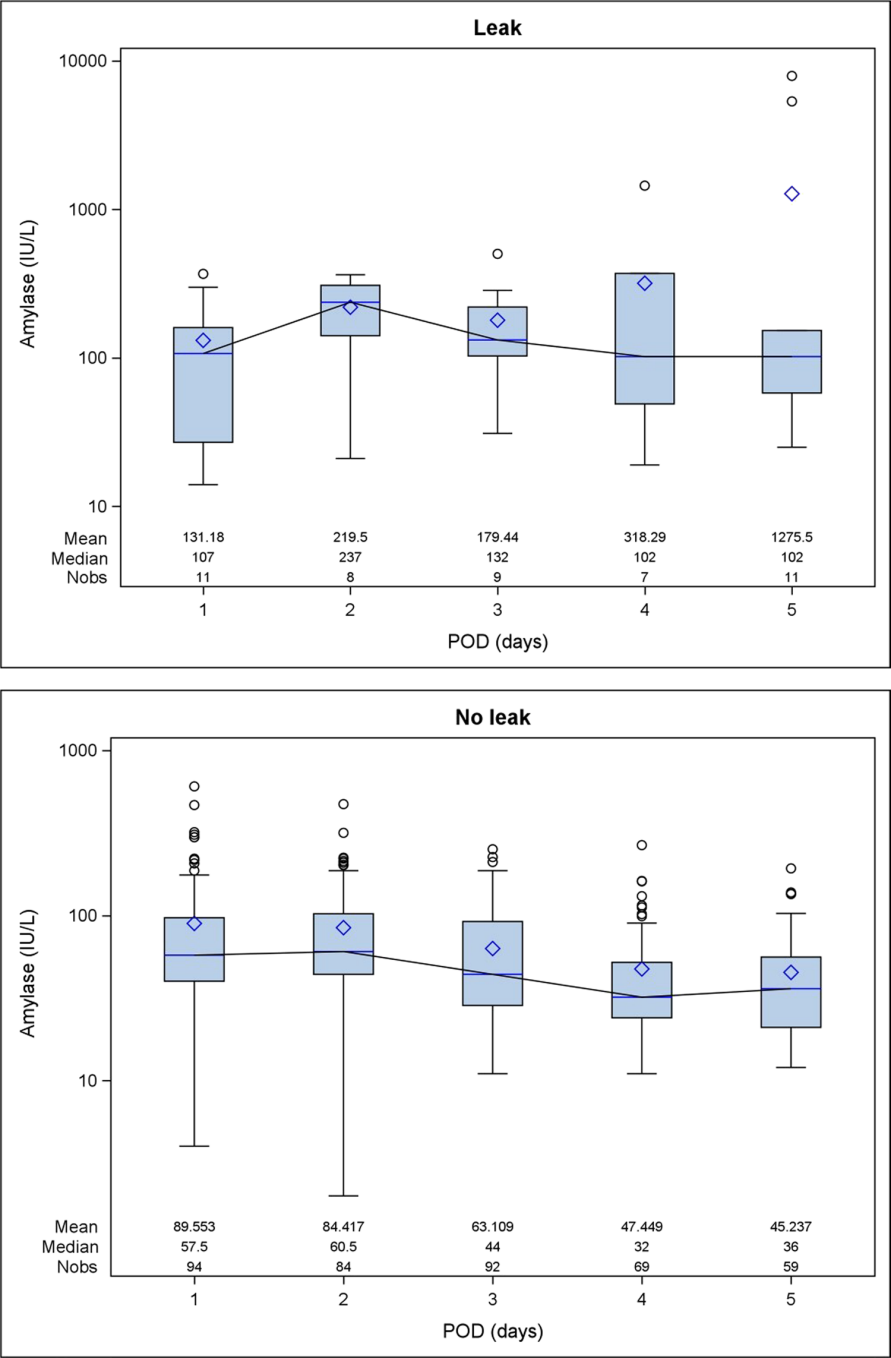
Postoperative variability of pleural drain amylase levels in the patient groups (no leak vs leak). POD, postoperative day

The CRP values showed a decreasing trend from POD 3 to 7 in the no leak group; conversely, CRP values remained almost steady and significantly higher until POD 5 in the leak group (Fig. 2). The peak median value was reached on POD 7. There was a statistically significant association between serum CRP levels and the presence of anastomotic leakage in POD 3, 5, and 7 (Table 2). Likewise, pleural drain amylase values were significantly higher in the leak group, reaching the median peak on POD2, and there was a statistically significant association between pleural amylase concentrations and the presence of leak (Fig. 3).

**Table 2:** Median postoperative values of serum C-reactive protein (CRP) and pleural drain amylase. Values are expressed as median and IQR

	POD	no. pts	LEAK (*n* = 12)	no. pts	NO LEAK (*n* = 109)	*p*
Serum CRP (mg/dL)	0	5	0.9 (1.0)	29	0.4 (1.0)	0.82
	3	9	23.1 (14.4)	103	12.5 (7.8)	0.007
	5	12	21.4 (11.9)	107	7.8 (6.0)	< 0.0001
	7	10	29.3 (15.5)	94	5.9 (5.3)	< 0.0001
Pleural drain amylase (UI/L)	1	11	107.0 (133.0)	94	57.5 (57.0)	0.17
	2	8	237.0 (167.0)	84	60.5 (58.5)	0.004
	3	9	132.0 (117.0)	92	44.0 (63.5)	0.002
	4	7	102.0 (322.0)	69	32.0 (28.0)	0.009
	5	11	102.0 (95.0)	59	36.0 (35.0)	0.0002

**Fig. 4:**
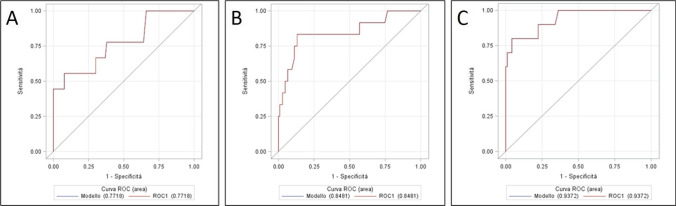
Serum C-reactive protein ROC curves for postoperative day 3 (**A**), 5 (**B**), and 7 (**C**). POD, postoperative day

Table 3 and Fig. 4 show the logistic regression models and the ROC curves analysis of serum CRP levels, which allowed the identification of the best cutoff values. In POD 3, the area under the curve (AUC) with the relative standard error (SE) was 0.772 ± 0.094, resulting in moderate accuracy in predicting anastomotic leakage. A CRP cutoff of 22.5 mg/dL demonstrated the best sensitivity (56%) and specificity (92%). As for the CRP values in POD 5 to 7, the AUC reached even higher accuracy, 0.848 ± 0.073 and 0.937 ± 0.040, respectively. This indicates that the practice of monitoring serum CRP levels is moderately accurate in predicting anastomotic leak on POD 3 and 5 and highly accurate on POD 7. It should be noted, however, that most leaks had already been diagnosed by POD 7. The likelihood ratio (LR) confirms the moderate utility of the test in predicting anastomotic leak. Moreover, the high negative predictive value (PV −) makes the serum CRP an effective indicator to exclude anastomotic leakage.

**Table 3:** Logistic regression analysis for serum C-reactive protein

POD	AUC ± SE	Cutoff	Sn	Sp	LR +	LR −	PV +	PV −	*p value*
3	0.772 ± 0.094	22.5	0.56	0.92	7.0	0.48	0.36	0.93	0.01^(*)^
5	0.848 ± 0.073	15.7	0.83	0.87	6.4	0.20	0.40	0.98	
7	0.937 ± 0.040	15.4	0.80	0.96	20	0.21	0.62	0.96	

*POD* postoperative day, *AUC* area under the curve, *SE* standard error, *Sn* sensitivity, *Sp* specificity, *LR* likelihood ratio, *VP* predictive value

^(*)^Comparison: CRP POD3-POD5, *p* = 0.08; CRP POD3-POD7, *p* = 0.008; CRP POD5-POD7, *p* = 0.38

**Fig. 5:**
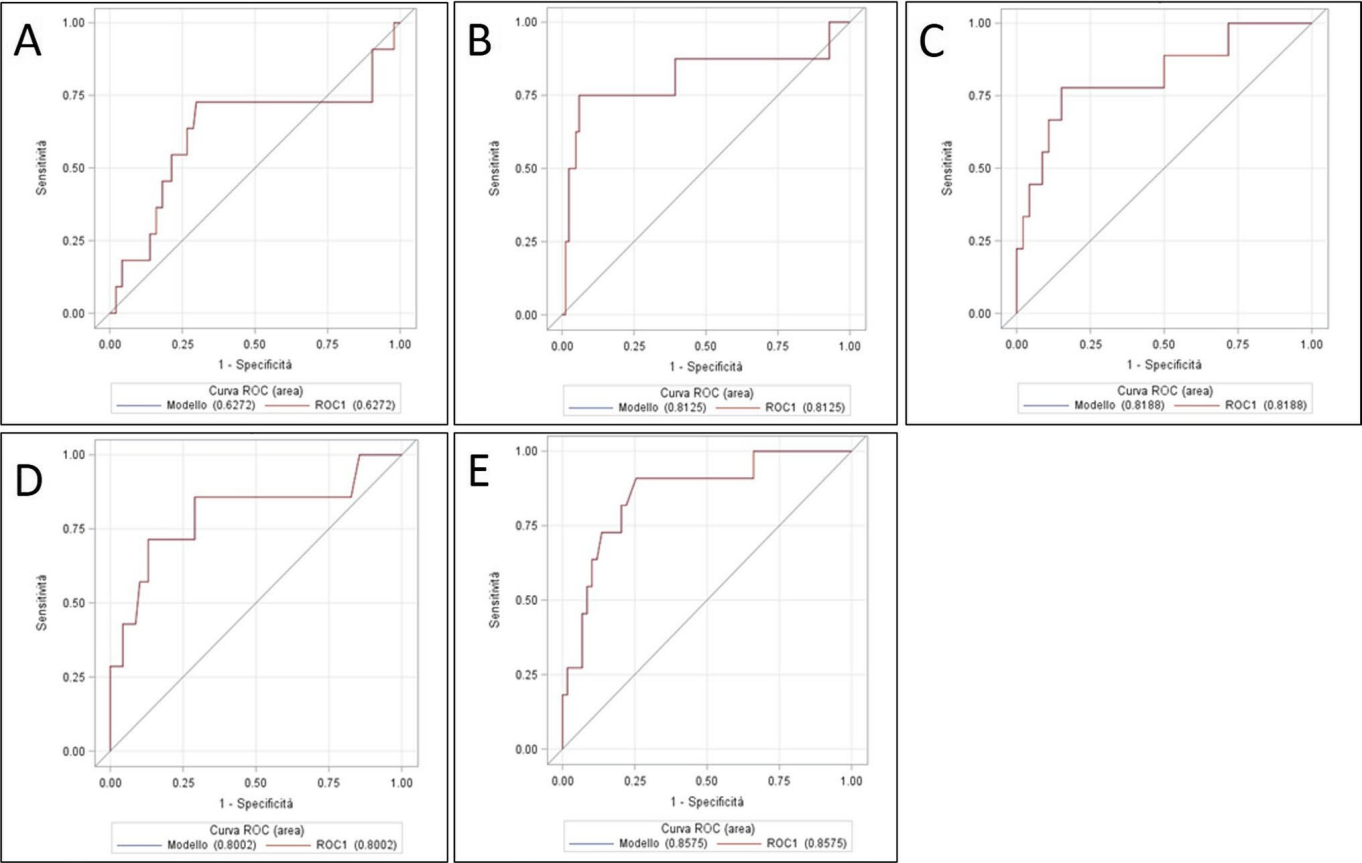
Pleural drain amylase ROC curves for postoperative day 1 (**A**), 2 (**B**), 3 (**C**), 4 (**D**), and 5 (**E**). POD, postoperative day

Table 4 and Fig. 5 illustrate outcomes relating logistic regression models and ROC curves analysis for pleural drain amylase. The AUC reached good levels of accuracy in all but the first POD. The best cutoff (209 IU/L) was on POD 2, with sensitivity and specificity values of 75% and 94%, respectively. The likelihood ratios and the predictive values (+ LR = 12.5, − LR = 0.27, PV +  = 0.50, PV −  = 0.94) indicate that the practice of measuring pleural amylase on POD 2 is moderately useful for early diagnosis of anastomotic leakage. The overall hospital cost per patient of pleural drain amylase sampling was about half compared to the cost of serum CRP testing (8.7 vs 17.4 euros). Comparison of ROC curves of CRP and amylase in day 3 and day 5 was not statistically significant (POD 3: CRP AUC = 0.77 ± 0.09; amylase AUC = 0.82 ± 0.09, *p* = 0.79, POD 5: CRP AUC = 0.85 ± 0.07; amylase AUC = 0.86 ± 0.06, *p* = 0.14).

**Table 4:** Logistic regression analysis for pleural drain amylase

POD	AUC ± SE	Cutoff	Sn	Sp	LR +	LR −	PV +	PV −	*p value*
1	0.627 ± 0.113	92	0.73	0.70	2,4	0.39	0.21	0.95	0.02^(*)^
2	0.813 ± 0.116	209	0.75	0.94	12.5	0.27	0.50	0.94	
3	0.819 ± 0.086	101	0.78	0.85	5.2	0.26	0.32	0.95	
4	0.800 ± 0.115	82	0.71	0.87	5.4	0.33	0.67	0.94	
5	0.858 ± 0.063	52	0.91	0.75	3.6	0.12	0.66	0.98	

*POD* postoperative day, *AUC* area under the curve, *SE* standard error, *Sn* sensitivity, *Sp* specificity, *LR* likelihood ratio, *VP* predictive value

^(*)^Comparison: amylase POD 1–2, *p* = 0.24; amylase POD 1–3, *p* = 0.30; amylase POD 1–4, *p* = 0.47; amylase POD 1–5, *p* = 0.14

## Discussion

This study shows that both pleural drain amylase and serum CRP after esophagectomy represent effective strategies for early detection of intrathoracic anastomotic dehiscence. This may translate into exclusion of patients at risk from the enhanced postoperative recovery pathway and may impact clinical practice by preventing the catastrophic consequences of anastomotic leakage and by reducing hospital costs. Anastomotic leakage after esophagectomy is associated with a significant increase in morbidity and mortality, and the time to diagnosis of the leak is the most influential factor in terms of prognosis [2]. In the present study, the contrast swallow study reached a diagnostic accuracy rate of only 50%.

Recommendations regarding early removal of drains and early oral feeding following esophagectomy are based on a low level of evidence, and no gold standards for predicting and diagnosing anastomotic leaks have been established yet. In fact, no studies have conclusively proven that perianastomotic drains help in the detection of leaks and that early feeding after esophagectomy is safe [10, 11].

It has previously been shown that CRP values > 18.1 mg/dL on POD 3 (Sn = 63%, Sp = 74%) indicate the need to perform second-level examinations to exclude or confirm the presence of anastomotic leakage. Conversely, CRP < 8.3 mg/dL on POD 5 (Sn = 89%, Sp = 60%) may avoid further radiological imaging and allow early oral feeding [12]. A systematic review found that CRP values lower than 17.6 mg/dL on POD 3 and 13.2 mg/dL on POD 5, combined with reassuring clinical and radiological findings, may exclude the presence of anastomotic leakage [4]. In the present study, the − LR of 0.20 on POD 5 confirms the moderate utility of CRP testing in excluding anastomotic leak. However, CRP is a non-specific biomarker of severe systemic inflammation in response to the severity of surgical trauma, blood loss, and duration of surgery, and can be abnormal even in patients who do not develop an anastomotic leak.

Sampling of pleural drain amylase has previously been proposed as a diagnostic tool to identify patients with anastomotic dehiscence [5]. In a retrospective study, chest-tube amylase levels were measured from POD 1 to 5. Amylase levels were significantly higher in patients with anastomotic leakage (*p* = 0.003). Cutoffs were then identified for the first 3 postoperative days showing high specificity (94.0–95.7%) but low sensitivity (21.4–35.7%) [13].

One of the largest studies including 146 patients undergoing esophagectomy and gastric conduit reconstruction with cervical or intrathoracic anastomosis showed that it is unlikely for a patient to develop anastomotic leak if drain amylase levels are below 38 IU/L in POD 4, whereas a value greater than 250 IU/L is almost always associated with leak. Moreover, drain amylase sampling showed greater accuracy than contrast esophagram in POD 4. However, only 17% of patients in this study underwent an intrathoracic anastomosis and it remains unclear whether amylase values are equally accurate for both cervical and intrathoracic leaks [14].

Another study including 80 patients evaluated the prognostic value of chest-tube amylase compared with serum CRP [15]. The authors used a CRP cutoff of 30 mg/dL, while in the first three PODs, a drain amylase cutoff of 335 IU/L showed sensitivity and specificity of 75% and 98%, respectively (versus 75% and 85% of serum CRP). Pleural drain amylase proved to be more accurate than serum CRP in predicting early anastomotic leak; moreover, the diagnosis was anticipated by 24 h compared to serum CRP. However, a limitation of this study is that only 4 early anastomotic leakages were identified.

 A recent systematic review investigating a total of 24 different biomarkers for early detection of upper gastrointestinal leaks found that the AUC for amylase varied from 0.70 to 0.81, indicating a good diagnostic accuracy with especially high negative predictive value. The authors concluded that the distance of the drain to the anastomosis may influence reported amylase levels, and that no single biomarker can predict anastomotic leak with absolute certainty, thereby suggesting that combined scores of biomarkers offer superior diagnostic accuracy [16].

In the present study, drain amylase kinetics demonstrated higher predictive value in detecting early anastomotic leak (*p* < 0.05) from POD 2 compared to serum CRP. In contrast with previous studies [17, 18], we systematically irrigated the pleural cavity before closing the chest to make amylase samplings reliable, and we chose POD 2 to compare amylase measurements in the leak and in the no leak group. The logistic regression model for pleural drain amylase showed a good diagnostic accuracy since POD 2 (AUC = 0.813) and the cutoff of 209 IU/L was associated with high sensitivity and specificity values (75% and 94%, respectively). Our data indicate that it is unlikely that a patient will develop an anastomotic leakage if pleural amylase concentrations are lower than 52 IU/L on POD 5. Furthermore, values higher than 209 IU/L starting from POD 2 were significantly associated with the presence of leak, and the − LR of 0.1 indicate moderate risk. Therefore, if these values are supported by suggestive clinical findings, second-level investigation including CT scan or endoscopic evaluation should be recommended before allowing oral feeding. However, we recommend not to interpret a single amylase value on a specific day, but rather the kinetics of amylase levels. By doing so, even if residual contaminants are present in the pleural cavity, the concentration of amylase would decrease in the no leak group and rise in the leak group, suggesting the need to perform second-level diagnostic tests. Moreover, our data confirm that pleural drain amylase sampling is a low-cost test with the potential to alert of the risk of anastomotic leakage up to 24 h earlier than serum CRP [15, 16].

Major limitations of this study are the retrospective design, the rather limited sample size, and the single-center setting. Selection and confounding bias due to unmeasured or unmeasurable factors cannot be excluded. However, the patient population was homogeneous regarding the surgical approach and only patients who received an intrathoracic anastomosis were included. A prospective multicenter study is required to allow the identification of reliable cutoff values and to validate the effectiveness of pleural fluid amylase sampling after esophagectomy,.

## Conclusions

Serial sampling of pleural fluid drain amylase is a simple, low-cost and effective method that may allow earlier prediction of esophago-gastric anastomotic leakage compared to serum CRP. This could optimize the postoperative recovery pathway by excluding from early feeding patients at risk for intrathoracic anastomotic leakage. Prospective studies are needed to test the hypothesis that combining pleural drain amylase and serum CRP may increase diagnostic accuracy and clinical utility.

## Data Availability

The data presented are available on request from the corresponding author.

## References

[1] Moon SW, Kim J, Cho DG, Park JK (2019). Early detection of complications: anastomotic leakage. J Thorac Dis.

[2] Grimminger P, Goense L, Gockel I (2018). Diagnosis, assessment, and management of surgical complications following esophagectomy. Ann New York Acad Sci.

[3] Cools-Lartigue J, Andalib A, Abo-Alsaud A (2014). Routine contrast esophagram has minimal impact on the postoperative management of patients undergoing esophagectomy for esophageal cancer. Ann Surg Oncol.

[4] Aiolfi A, Asti E, Rausa E, Bonavina G, Bonitta G, Bonavina L (2018). Use of C-reactive protein for the early prediction of anastomotic leak after esophagectomy: systematic review and Bayesian meta-analysis. PLoS ONE.

[5] Machens A, Busch C, Bause H, Izbicki JR (1996). Gastric tonometry and drain amylase analysis in the detection of cervical oesophagogastric leakage. Br J Surg.

[6] Miller DL, Helms GA, Mayfield WR (2018). Evaluation of esophageal anastomotic integrity with serial pleural amylase levels. Ann Thorac Surg.

[7] Bonavina L, Asti E, Sironi A, Bernardi D, Aiolfi A (2017). Hybrid and total minimally invasive esophagectomy: how I do it. J Thorac Dis.

[8] Asti E, Bernardi D, Bonitta G, Bonavina L (2018). Outcomes of transhiatal and intercostal pleural drain after Ivor Lewis esophagectomy: comparative analysis of two consecutive patient cohorts. J Laparoendosc Adv Surg Tech.

[9] Dindo D, Demartines N, Clavien PA (2004). Classification of surgical complications: a new proposal with evaluation in a cohort of 6336 patients and results of a survey. Ann Surg.

[10] Jamel S, Tukanova K, Markar SR (2019). The evolution of fast track protocols after oesophagectomy. J Thorac Dis.

[11] Low DE, Allum W, De Manzoni G (2019). Guidelines for perioperative care in esophagectomy: enhanced recovery after surgery (ERAS) society recommendations. World J Surg.

[12] Asti E, Bonitta G, Melloni M (2018). Utility of C-reactive protein as predictive biomarker of anastomotic leak after minimally invasive esophagectomy. Langenbeck's Arch Surg.

[13] Berkelmans G, Kouwenhoven EA, Smeets B (2015). Diagnostic value of drain amylase for detecting intrathoracic leakage after esophagectomy. World J Gastroenterol.

[14] Perry Y, Yowe C, Kwong J (2015). Serial drain amylase can accurately detect anastomotic leak after esophagectomy and may facilitate early discharge. Ann Thorac Surg.

[15] Giulini L, Dubecz A, Solymosi N (2019). Prognostic value of chest-tube amylase versus C-reactive protein as screening tool for detection of early anastomotic leaks after Ivor Lewis esophagectomy. J Laparoendosc Adv Surg Tech.

[16] De Mooij CM, van den Brink MM, Merry A, Tweed T, Stoot J (2019). Systematic review of the role of biomarkers in predicting anastomotic leakage following gastroesophageal cancer surgery. J Clin Med.

[17] Baker EH, Hill JS, Reames MK (2015). Drain amylase aids detection of anastomotic leak after esophagectomy. J Gastrointest Oncol.

[18] Yu WS, Jung J, Shin H (2019). Amylase level in cervical drain fluid and anastomotic leakage after cervical oesophagogastrostomy. Eur J Cardio-Thor Surg.

